# The correlation between nutrition index scores and nutrition status of preschool children in a district of Shanghai

**DOI:** 10.3389/fnut.2026.1746871

**Published:** 2026-04-13

**Authors:** Hangwei Wang, Yiyao Zhou, Zhongying Lu, Xiaoyan Gong, Shuo Zhang, Wennan He, Weili Yan, Tian Qian

**Affiliations:** Children's Hospital of Fudan University, Shanghai, China

**Keywords:** BMI Z-score, family-centered intervention, nutrition quotient for preschoolers (NQ-P), preschool children, quasi-experimental study

## Abstract

This quasi-experimental pre-post study aimed to evaluate factors influencing children’s nutritional quality using the Nutrition Quotient for Preschoolers (NQ-P) and assess the impact of a targeted family-centered intervention on physical development and dietary behaviors. A total of 1,794 children (mean age: 4.78 ± 0.97 years; 53% boys) were included in the cross-sectional baseline analyses, with a convenience subsample of 163 children participating in a three-month nutritional intervention. Baseline results indicated no significant gender- or age-related differences in NQ-P scores (*p* > 0.05). However, significant positive correlations were observed between higher NQ-P scores and factors such as parental dietary responsibility, frequent family meals, and outdoor exercise (*p* < 0.05). Total NQ-P and moderation domain scores were lower in overweight/obese children (BMIZ = 1–3) than in their normal-weight peers (*p* < 0.05 and *p* < 0.001, respectively). Post-intervention, significant improvements were noted: the mean BMI decreased from 15.79 ± 3.03 to 15.40 ± 1.90 (*p* < 0.001), with a notable reduction in overweight/obesity prevalence from 23.9 to 18.4% (*p* < 0.01). Total NQ-P scores increased from 67.68 ± 8.68 to 68.74 ± 9.02 (*p* < 0.001), driven by enhancements in the balance and moderation domains across all BMIZ groups (*p* < 0.05). The intervention stabilized BMI in normal-weight children and improved nutritional outcomes in underweight and overweight subgroups. These findings suggest the potential effectiveness of family-centered dietary management and structured physical activity in enhancing children’s nutritional quality, particularly for those with elevated BMIZ. Tailored interventions based on individual BMIZ classifications are recommended to optimize health outcomes. This study provides evidence-based insights for designing targeted strategies to address childhood malnutrition and obesity.

## 1 Introduction

The preschool period is a critical stage for children’s physical development, during which dietary intake and growth status profoundly impact their future physical and mental health (1, 2). Research indicates that nutritional habits formed during childhood often persist into adulthood (3). Preschool children with malnutrition face an increased risk of developing chronic diseases. At the same time, unhealthy dietary behaviors are closely associated with rapid weight gain, increased risk of childhood obesity, and the development of obesity and cardiovascular diseases in adulthood (4, 5). Therefore, focusing on the dietary behaviors of preschool children and ensuring balanced nutritional intake is crucial for promoting their comprehensive and healthy development.

However, with globalization and lifestyle changes, dietary behavior issues among preschool children have become increasingly prominent. The prevalence of malnutrition, obesity, and other diet-related diseases has been rising annually, posing a significant challenge to global public health. The United Nations Children’s Malnutrition Assessment Organization (6) reports that one-third of children worldwide experience malnutrition, comprising 149.2 million stunted, 45.4 million wasted, and 38.9 million overweight children under five years (7). In China, the 2020 report from the National Health Commission revealed that the prevalence of stunting among children under six years of age was 4.8%, wasting was 2.0%, overweight was 6.8%, and obesity was 3.6% (8). This indicates that approximately 17.4% of children in this age group suffer from malnutrition or overnutrition, both of which may adversely affect their physical development. Furthermore, the prevalence of overweight and obesity among Chinese children varies by socioeconomic status (SES), with higher rates observed among children from higher SES backgrounds (9). For example, in Shanghai, a more economically developed region, the prevalence of malnutrition among children was 6.66%, while the rates of overweight and obesity were 17.81 and 13.25%, respectively (10).

Given the profound impact of dietary behaviors on preschool children’s current nutritional status, health, and future disease risks, it is necessary to conduct scientific research and systematic assessments to analyze their dietary behavior characteristics and related influencing factors (11). The NQ-P (Nutrition Quotient for Preschoolers) scale is a specialized tool for assessing preschool children’s dietary quality and behavior-related habits completed by parents. This scale demonstrates simplicity, efficiency, comprehensiveness, ease of implementation, and guardian-friendly design. Numerous studies have widely recognized its scientific validity and practicality (12, 13). Yan et al. (14) translated the scale into a Chinese version and validated its reliability and validity, providing a reliable tool for related research.

The preschool period represents a critical window for nutritional intervention. Early investment in children’s nutrition improves their health and reduces the economic burden of diet-related non-communicable diseases (15). Therefore, identifying and addressing issues in children’s dietary behaviors through scientific assessments and targeted interventions is of great significance for fostering healthy nutritional habits in preschool children.

Based on the above background, this study focuses on preschool children’s dietary behavior characteristics and influencing factors. It aims to establish healthy dietary habits and patterns through systematic assessment and intervention, providing scientific evidence and practical support for healthy growth.

## 2 Study design and methods

### 2.1 Study design and population

This quasi-experimental study with a pre-post design was conducted among parents of preschool children aged 3 to 6.5 years attending public kindergartens in Minhang District, Shanghai. A Chinese version of the NQ-P questionnaire was administered to collect data. Participations were voluntary, and we distributed the questionnaires to kindergartens via QR codes. Kindergarten administrators facilitated the distribution, and we collected data through a backend system. We defined valid questionnaires as those with fully completed items and realistic demographic information. After the first survey, we recruited a convenience subsample from the baseline cohort for a three-month nutritional intervention program. Recruitment was conducted through kindergarten-level invitations; parents who voluntarily agreed to participate in the intervention were enrolled (*n* = 163). No randomization or matching procedures were applied. Following the intervention, a second survey was conducted using the same methodology. Ethical approval was obtained from the Ethics Committee of Children’s Hospital of Fudan University (approval number: 2025-184). Written informed consent was waived due to the observational nature of the survey component; however, the QR-code-based survey included a mandatory consent-to-participate landing page, where parents were informed about the study purpose, data confidentiality, and their right to withdraw at any time before proceeding with the questionnaire.

### 2.2 Questionnaire survey

The questionnaire comprised two sections: general demographic information about the children and their parents and the NQ-P scale.

### 2.3 General demographic characteristics

Demographic data included the child’s age, gender, height, and weight; parental education levels; primary household dietary decision-maker; annual household income; daily number of family meals; presence of significant health symptoms in the child over the past month; parental recording of the child’s dietary habits; provision of extra meals; and daily outdoor activity time.

### 2.4 NQ-P scale

The NQ-P scale, a Chinese version of the Nutrition Quotient for Preschoolers (14), the Cronbach’s *α* coefficients for the total score and individual domains ranged from 0.706 to 0.774, systematically assessed children’s dietary behaviors. This self-administered scale includes 14 items across three domains:

Balance Domain (5 items): evaluate dietary diversity and nutritional balance through vegetable intake (per meal) and consumption frequency of pure milk, legumes/soy products, poultry/livestock meat, and fish.

Moderation Domain (4 items): focus on the consumption frequency of processed meats, fast food, sweet/high-fat baked goods, and processed beverages.

Environment Domain (5 items): analyze the dietary environment and behavioral habits, including breakfast regularity, focused mealtime behavior (e.g., avoiding fidgeting), family support for healthy eating, handwashing before meals, and daily screen time.

Raw scores for each domain were linearly standardized to a 0–100 scale. The total score was calculated using a weighted formula:


$$\begin{array}{c} \text{Total Score}=(\text{Balance}\times 0.45)+(\text{Moderation}\times 0.30)\\ +(\text{Environment}\times 0.25) \end{array}$$

Total scores range from 0 to 100, with higher scores indicating healthier dietary behaviors.

Detailed distributions of NQ-P scores by gender and age groups, item-specific correlation analyses between NQ-P scale scores and BMI Z-scores, and post-hoc comparisons among different physical development groups are provided in Supplementary Tables 1–3.

### 2.5 Physical measurements

We calculated the children’s Body Mass Index (BMI) using their height and weight data from the questionnaire. BMI-for-age Z-scores (BMIZ) were calculated using the WHO Anthro Survey Analyser (version 3.2.2) based on the WHO Child Growth Standards (for children <5 years) and WHO Growth Reference 2007 (for children 5–19 years) (16, 17).

## 3 Nutritional intervention

The intervention comprised two components:

### 3.1 Nutritional science lecture

A certified pediatric nutritionist (with a Master’s degree in clinical nutrition and >10 years of experience) delivered four nutrition education sessions per month during the three-month intervention period (12 sessions in total). Sessions were delivered in a hybrid format (in-person at kindergartens and simultaneously live-streamed online) and covered balanced meal planning, age-appropriate portion sizes, reduction of sugar-sweetened beverages, and promotion of fruit and vegetable intake. Each session lasted approximately 45 min, including an interactive Q&A segment. Attendance was documented via the online platform for implementation monitoring.

### 3.2 Healthy habit tracking program

The healthy habit tracking program was designed with reference to UNICEF’s “Know Your Food” initiative (18). Parents were encouraged to complete daily tasks (e.g., replacing snacks with fruit or nuts, avoiding sugary drinks, encouraging water intake, and including vegetables in daily meals) and upload punch-card records to the mini-program as text or photos. Researchers performed regular backend reviews of submitted records and provided follow-up reminders.

Detailed demographic characteristics of the children who participated in the intervention study, as well as detailed changes in BMI and BMIZ before and after the intervention, are provided in Supplementary Tables 7, 8.

### 3.3 Quality control and sensitivity analysis

Quality control measures were implemented throughout data collection and analysis. All questionnaires were reviewed for completeness and internal consistency before analysis. Two trained researchers independently checked exported data and reconciled discrepancies through consensus.

To assess the robustness of our findings, we conducted sensitivity analyses excluding children who reported obvious symptoms of discomfort in the past month (*n* = 129, 7.2% of baseline sample). These symptoms were parent-reported and included gastrointestinal complaints (e.g., vomiting, diarrhea, abdominal pain), febrile illness, respiratory infections, or any other acute conditions that may have temporarily altered the child’s dietary intake or appetite, resulting in an analytical sample of 1,665 children. This exclusion was performed to minimize potential confounding effects of acute health conditions on dietary behaviors and nutritional assessments.

### 3.4 Statistical analysis

Data were analyzed using SPSS Statistics (version 23.0). Descriptive statistics, including means and standard deviations (M ± SD), were calculated for demographic characteristics, NQ-P scores, and other continuous variables. The normality of data distribution was assessed using the Shapiro–Wilk test; given the large sample size (*n* = 1,794), parametric tests were considered robust to minor deviations from normality based on the Central Limit Theorem. For comparisons between groups, independent samples t-tests or one-way analysis of variance (ANOVA) were applied as appropriate, followed by Bonferroni-adjusted post-hoc tests when significant differences were detected. Pearson correlation coefficients were calculated to examine bivariate associations between NQ-P scores and demographic variables, as well as between NQ-P domain scores and BMI Z-scores. To account for potential confounding, multivariable linear regression analyses were performed with total NQ-P score as the dependent variable, adjusting for child age, gender, parental education, household income, and dietary responsibility status. Effect sizes were reported as Cohen’s d for paired comparisons and partial eta-squared (η^2^p) for ANOVA models; 95% confidence intervals (CIs) were provided for key estimates. For the intervention analysis, paired samples t-tests were used to compare pre- and post-intervention measurements within the same participants (*n* = 163). Missing data were minimal (<2% for any variable); complete-case analysis was employed as the primary approach. Sensitivity analyses were conducted excluding participants reporting obvious symptoms of discomfort in the past month (*n* = 129). The consistency of findings between the primary analysis (*n* = 1,794) and sensitivity analysis (*n* = 1,665) was evaluated to assess robustness. Statistical significance was set at *p* < 0.05 (two-tailed) for all analyses.

## 4 Results

### 4.1 Baseline characteristics of the study population

A total of 1,794 valid questionnaires (94.2%) were analyzed, with children averaging 4.78 ± 0.97 years old (53% boys). The mean BMI Z-score was 0.14 ± 1.03, with 25.5% classified as overweight/obese. Mothers primarily completed questionnaires (76.7%) and were the main dietary caregivers (70.3%). Most parents (93.7%) held college degrees or higher, and 78.1% of families consisted of four or more members. Additionally, 77.5% provided extra meals, and 94.6% ensured over 30 min of daily physical activity (see Table 1).

**Table 1:** Baseline characteristics of the study population *n* (%).

Variables	Overall (*n* = 1794)	Boy (*n* = 950)	Girl (*n* = 844)
Age (one year old, M ± S)	4.78 ± 0.97	4.76 ± 0.99	4.81 ± 0.97
2 ~ <3	2 (0.1)	1 (0.0)	1 (0.1)
~ < 4	183 (10.2)	104 (5.1)	79 (7.5)
~ < 5	523 (29.2)	286 (14.0)	237 (22.6)
~ < 6	584 (32.6)	296 (14.5)	288 (27.4)
~ < 7	499 (27.8)	261 (12.8)	238 (22.7)
~ < 8	3 (0.2)	2 (0.1)	1 (0.1)
BMI (M ± S)	15.68 ± 2.03	15.83 ± 2.04	15.51 ± 2.00
BMI Z score (M ± S)	0.14 ± 1.03	0.13 ± 1.06	0.15 ± 1.00
−3	36 (2.0)	20 (2.1)	16 (1.9)
−2	62 (3.5)	39 (4.1)	23 (2.7)
−1	191 (10.6)	99 (10.4)	92 (10.9)
0	1,051 (58.5)	554 (58.2)	497 (58.8)
1	291 (16.2)	147 (15.4)	144 (17.0)
2	120 (6.7)	68 (7.1)	52 (6.2)
3	46 (2.6)	25 (2.6)	21 (2.5)
Only child			
Yes	951 (52.9)	514 (54.0)	437 (51.7)
No	846 (47.1)	438 (46.0)	408 (48.3)
Height (cm, M ± S)	112.56 ± 8.39	112.91 ± 8.44	112.16 ± 8.32
Weight (kg, M ± S)	19.96 ± 3.95	20.30 ± 4.10	19.58 ± 3.74
Maternal education			
High school and below	120 (6.7)	73 (7.7)	47 (5.6)
Associate or undergraduate	1,178 (65.6)	615 (64.6)	563 (66.6)
Graduate student	360 (20.0)	197 (20.7)	163 (19.3)
Not to be revealed	139 (7.7)	67 (7.0)	72 (8.5)
Paternal education			
High school and below	113 (6.3)	67 (7.0)	46 (5.4)
Associate or undergraduate	1,116 (62.1)	591 (62.1)	525 (62.1)
Graduate student	423 (23.5)	223 (23.4)	200 (23.7)
Not to be revealed	145 (8.1)	71 (7.5)	74 (8.8)
Whether to be responsible for the diet			
Yes	1,263 (70.3)	670 (70.4)	593 (70.2)
No	534 (29.7)	282 (29.6)	252 (29.8)
Fill in the relationship between the person and the child			
Mother	1,378 (76.7)	740 (77.7)	638 (75.5)
Father	394 (21.9)	200 (21.0)	194 (23.0)
Grandparent or maternal grandparent	24 (1.3)	12 (1.3)	12 (1.4)
Other	1 (0.1)	0 (0.0)	1 (0.1)
Annual household income (10,000/year)			
<5	32 (1.8)	20 (2.1)	12 (1.4)
~ 10	62 (3.5)	26 (2.7)	36 (4.3)
~ 15	100 (5.6)	55 (5.8)	45 (5.3)
~ 20	108 (6.0)	63 (6.6)	45 (5.3)
>20	720 (40.1)	375 (39.4)	345 (40.8)
Not to be revealed	775 (43.1)	413 (43.4)	362 (42.8)
Number of daily family meals			
≤3	393 (21.9)	214 (22.5)	179 (21.2)
4	653 (36.3)	348 (36.6)	305 (36.1)
5	518 (28.8)	269 (28.3)	249 (29.5)
≥6	233 (13.0)	121 (12.7)	112 (13.3)
Whether the child has obvious symptoms of discomfort in the past month			
Yes	129 (7.2)	81 (8.5)	48 (5.7)
No	1,668 (92.8)	871 (91.5)	797 (94.3)
Parents take the initiative to record their children’s eating and drinking.			
Never	654 (36.4)	355 (37.3)	299 (35.4)
Occasionally	635 (35.3)	320 (33.6)	315 (37.3)
Sometimes	286 (15.9)	149 (15.7)	137 (16.2)
Often	173 (9.6)	100 (10.5)	73 (8.6)
All the time	49 (2.7)	28 (2.9)	21 (2.5)
Extra meal or not			
Extra meals daily	216 (12.0)	129 (13.6)	87 (10.3)
Occasional extra meals	1,183 (65.8)	622 (65.3)	561 (66.4)
Never extra meals	398 (22.1)	201 (21.1)	197 (23.3)
Time of day for outdoor exercise			
< 30 min	98 (5.5)	44 (4.6)	54 (6.4)
30 min.–an hour	808 (45.0)	426 (44.7)	382 (45.2)
1 to 2 h	682 (38.0)	357 (37.5)	325 (38.5)
≥2 h	209 (11.6)	125 (13.1)	84 (9.9)

### 4.2 Sensitivity analysis results

After excluding children with obvious discomfort symptoms in the past month, the analytical sample comprised 1,665 children (869 boys, 796 girls) with a mean age of 5.20 ± 0.95 years. The sensitivity analysis demonstrated consistent findings with the primary analysis across all key outcomes: The correlation patterns between NQ-P scores and influencing factors remained stable. Parental dietary responsibility (r = −0.073, *p* < 0.05), daily family meals frequency (r = 0.057, *p* < 0.05), parental recording of children’s diet (r = 0.178, *p* < 0.01), and daily outdoor exercise time (r = 0.129, *p* < 0.01) continued to show significant associations with total NQ-P scores.

Similarly, the relationships between BMI Z-score and NQ-P domains persisted: moderation domain (r = −0.079, *p* < 0.01) and environment domain correlations remained significant. These consistent results across both the full sample and sensitivity analysis confirm the robustness of our findings. Detailed sensitivity analysis results are provided in Supplementary Tables 4–6.

### 4.3 Correlation analysis between scale score and each influencing factor

Table 2 reveals significant correlations between various factors and NQ-P scores. Specifically, the total NQ-P score was significantly associated with whether the individual was responsible for dietary management, the frequency of daily family meals, the presence of noticeable discomfort symptoms in children during the past month, parental initiative in recording children’s diets, and the duration of daily outdoor exercise.

**Table 2:** Correlation analysis between scale score and each influencing factor.

Variables	NQ-P total score	Balance domain	Moderation domain	Environmental domain
Gender	−0.001	0.005	−0.026	0.029
Age	−0.003	0.022	−0.028	0.026
BMI	−0.029	−0.021	−0.069**	0.015
BMI Z score	−0.034	−0.028	−0.067**	0.003
Be an only child	0.028	−0.013	0.007	0.048*
Height	−0.026	0.025	−0.006	0.049*
Weight	−0.039	0.007	−0.050*	0.046
Maternal education	0.014	−0.019	0.000	0.014
Paternal education	0.042	−0.009	0.001	0.037
Is responsible for the diet	−0.061**	0.054*	−0.046	−0.045
Fill in the human-child relationship	−0.023	−0.030	−0.025	−0.053*
Annual household income (10,000/year)	0.032	0.028	0.046	0.037
Number of daily family meals	0.049*	0.034	−0.008	0.035
Whether the child has obvious symptoms of discomfort in the past month	0.053*	0.003	−0.006	0.021
Parents take the initiative to record their child’s diet	0.193**	0.057*	0.058*	0.057*
Extra meal or not	−0.001	0.025	0.021	0.054*
Daily outdoor exercise time	0.127**	0.037	0.009	−0.007

*p < 0.05, statistically significant difference; **p < 0.01, statistically significant difference.

Similar trends were observed in the balance domain, which exhibited positive correlations with being responsible for diet and parental proactive recording of children’s diets, suggesting that these factors contribute to maintaining a balanced diet.

In the moderation domain, BMI, BMI z-score, and body weight showed negative correlations, while parental active recording of children’s diets demonstrated a positive correlation with this domain.

Within the environment domain, being an only child, height, family initiative in recording children’s diets, and the practice of consuming additional meals were positively correlated with the domain score. Conversely, interpersonal relationships with children exhibited a negative correlation with the environment domain.

### 4.4 Correlation between children’s physical development and NQ-P scale scores

Correlation analysis revealed significant associations between BMIZ and NQ-P domain scores. The moderation domain showed a negative correlation with BMIZ (r = −0.079, *p* < 0.01), indicating that children with higher BMI Z-scores had poorer dietary moderation behaviors. Specific behavioral items significantly associated with BMIZ included processed beverage consumption (r = 0.054, *p* < 0.05), remaining seated during meals (r = 0.065, *p* < 0.01), and daily screen time (r = 0.111, *p* < 0.01). These findings suggest that unhealthy eating behaviors and sedentary habits may contribute to increased risk of overweight in children. Detailed item-specific correlations are provided in Supplementary Table 3 (see Table 3).

**Table 3:** Correlation analysis between NQ-P scale items and BMIZ.

Domain	Item content	BMIZ
Balance	How many servings of vegetables does your child consume per meal on average in the past month? (1 serving = 1 small plate)	−0.017
	How often did your child consume dairy products (e.g., pure milk, formula milk) in the past month?	−0.034
	How often did your child consume soy products (e.g., soy milk, tofu, dried beans) in the past month?	0.006
	How often did your child consume poultry or livestock meat (e.g., pork, beef, chicken, duck) in the past month?	−0.017
	How often did your child consume fish in the past month?	−0.026
Moderation	How often did your child consume processed meats (e.g., ham, sausages) in the past month?	0.038
	How often did your child consume fast food (e.g., hamburgers, pizza) in the past month?	0.042
	How often did your child consume snacks (e.g., candy, chocolate) or desserts (e.g., cake, doughnuts) in the past month?	0.015
	How often did your child consume processed beverages in the past month?	0.054*
Environment	Does your child usually eat breakfast?	0.027
	Does your child remain seated and avoid fidgeting during meals?	0.065**
	Do you and your family help the child develop healthy eating habits?	−0.004
	Does your child wash hands before meals?	0.015
	How much time does your child spend daily on TV, mobile phones, tablets, or video games?	0.111**

*p < 0.05, statistically significant difference; **p < 0.01, statistically significant difference.

### 4.5 Follow-up population characteristics

In the original study cohort (*n* = 1,794), 163 children participated in the follow-up intervention. After applying sensitivity analysis exclusion criteria, 156 children from the analytical sample (*n* = 1,665) participated in the intervention study. Comparison of baseline characteristics between the intervention group and non-participants showed no significant differences in age, gender distribution, BMI Z-score, parental education levels, or family income (all *p* > 0.05), indicating minimal selection bias in the intervention sample (detailed comparisons in Supplementary Table 7) (see Table 4).

**Table 4:** Differences of BMI and BMIZ before and after intervention.

Variables	Pre-intervention	Post-intervention
BMI	15.79 ± 3.03	15.40 ± 1.90***
BMIZ = −3 ~ −1 (*n* = 30)	12.99 ± 0.74	13.96 ± 1.94**
BMIZ = 0 (*n* = 95)	15.22 ± 0.76	15.25 ± 1.45
BMIZ = 1 ~ 3 (*n* = 38)	19.28 ± 4.24	16.80 ± 1.88**
BMIZ	0.09 ± 1.15	−0.04 ± 1.00***
BMIZ = −3	5 (3.1)	5 (3.1)
BMIZ = −2	8 (4.9)	5 (3.1)
BMIZ = −1	16 (9.8)	24 (14.7)
BMIZ = 0	95 (58.3)	99 (60.7)
BMIZ = 1	25 (15.3)	22 (13.5)
BMIZ = 2	6 (3.7)	4 (2.5)
BMIZ = 3	8 (4.9)	4 (2.5)

**p < 0.01, statistically significant difference; ***p < 0.001, statistically significant difference.

### 4.6 Differences in BMI values and BMIZ ranges before and after intervention

After the intervention, children’s mean BMI dropped from 15.79 to 15.40 (*p* < 0.001), and BMIZ decreased from 0.09 to −0.04 (*p* < 0.001), indicating improved weight status. Underweight children (BMIZ −3 to −1) saw BMI increase (*p* < 0.05), while overweight/obese children (BMIZ 1 to 3) saw BMI decrease significantly (*p* < 0.01). Normal-weight children showed no BMI change. Nutritional performance (NQ-P) improved across all BMIZ groups (*p* < 0.001). Balance and moderation scores improved significantly, especially in underweight and overweight groups. Environmental domain scores improved except in underweight children. Overall, the intervention was associated with improvements in nutrition and BMI outcomes, with varying effects across initial BMIZ classifications. However, without a concurrent control group, the possibility that these changes reflect natural growth patterns, seasonal dietary variations, regression to the mean, or the Hawthorne effect cannot be ruled out.

## 5 Discussion

Family intervention in children’s growth and development and the role of family-based intervention strategies are essential in improving children’s nutritional status (19). This study explored the correlation between nutrition index scores and the nutritional status of preschool children in a district of Shanghai, revealing that gender and age were not significantly associated with NQ-P scores in this sample. While this may suggest relatively uniform dietary patterns among preschoolers in this urban Shanghai context, this finding should be interpreted cautiously given the relatively homogeneous socioeconomic composition of our sample (93.7% of parents held college degrees or higher, and 40.1% reported annual household incomes >200,000 RMB). Such socioeconomic homogeneity may attenuate gender- and age-related dietary differences that might otherwise be observed in more diverse populations. Additionally, family-related factors, such as parental dietary involvement and meal routines, were strongly associated with higher nutrition scores, emphasizing the crucial role of family in shaping children’s eating habits. The intervention was associated with improvements in nutritional status, particularly among overweight children, suggesting the potential value of structured, family-involved strategies in promoting healthier dietary behaviors. However, given the quasi-experimental pre-post design without a control group, these improvements should be interpreted with caution, as they cannot be definitively attributed to the intervention alone. To provide additional comprehensive details, this study includes distributions of nutrition quality scores by gender and age groups, item-specific correlations with BMI Z-scores, detailed post-hoc comparisons across physical development categories, and comprehensive demographic and physical development changes pre- and post-intervention as Supplementary Tables 1–8.

Our study found that NQ-P scores significantly correlate with children’s dietary habits, family food environment, and exercise patterns. Children whose parents take more responsibility for meals have higher family meal frequency, and longer outdoor activity time generally have higher NQ-P scores. Prior research has also shown that family meal patterns closely relate to children’s dietary quality (20), with parental involvement in meal decisions effectively improving children’s nutritional intake quality and reducing overweight and obesity risks. This observation aligns with prior research that maintained consistent IQ levels across different age groups of children (21). Similar research found that boys and girls show very similar trends in dietary patterns over time, suggesting gender differences in dietary patterns may not be significant (22).

Regarding weight status and NQ-P scores, overweight/obese children had lower NQ-P scores, particularly in the “moderation domain,” indicating potential issues with excessive calorie intake and uneven nutrient distribution. Previous studies assessing dietary quality indexes and children’s health outcomes found higher dietary quality associated with reduced obesity risk (23). One study developing a Chinese children’s dietary quality index found higher scores associated with lower BMI, especially in moderation (limiting high-sugar, high-fat foods) (24). Overweight/obese children also had lower “balance domain” scores, suggesting potentially unbalanced dietary structures with insufficient protein, dietary fiber, and micronutrient intake. Additionally, processed beverage consumption frequency (e.g., sugary drinks) positively correlated with BMIZ (r = 0.054, *p* < 0.05), indicating increased obesity risk, supported by Malik et al. (25) who found each additional daily serving of sugary beverages associated with a 0.07-kg/m^2^ BMI increase. In the environmental domain, the association between sitting behavior during meals and BMIZ (r = 0.065, *p* < 0.01) warrants nuanced interpretation: this positive correlation may reflect that children with higher BMI tend to be less physically active or fidgety rather than indicating that sitting still causes weight gain. Similarly, daily screen time (r = 0.111, *p* < 0.01) positively correlated with BMIZ, consistent with Robinson et al. (26) who reported that 60% of overweight incidence in 4-year-olds attributes to excessive television viewing.

Family dietary management and activity interventions were associated with improvements in children’s BMI and NQ-P scores. Post-intervention, mean BMI decreased from 15.79 to 15.40 (Cohen’s d = 0.15), and overweight/obesity prevalence declined from 23.9 to 18.4%. While the absolute BMI change of 0.39 kg/m^2^ and the NQ-P score increase of approximately 1 point may appear modest, these findings should be interpreted in clinical context. In growing preschoolers, even small shifts in BMI trajectory may have long-term relevance, as childhood nutrition and growth patterns are linked to later health outcomes (5, 23). Moreover, the 5.5 percentage-point reduction in overweight/obesity prevalence represents a meaningful shift at the population level. NQ-P scores also improved, especially in the balance and moderation domains. These findings indicate that structured family dietary management can support improvements in preschool children’s weight status and dietary quality. Previous studies have shown that school-only intervention components often yield limited effects unless family-level behavior change is incorporated (27, 28).

In contrast, family dietary interventions can maintain good health behavior over extended periods. Recent evidence strongly supports the effectiveness of family-based interventions. A 2023 cluster randomized controlled trial in South India demonstrated that home-based interventions significantly improved nutritional status in preschool children through caregiver involvement in nutritious diet preparation, leading to sustained healthy dietary practices within families (29). Additionally, a large-scale randomized clinical trial published in JAMA (2023) involving 452 children across four US settings showed that family-based behavioral treatment implemented in pediatric primary care settings produced significant improvements in childhood obesity outcomes over 24 months, with children in the intervention group showing a 6.21% greater reduction in percentage above median BMI compared to usual care (30). Recent evidence by Drapeau et al. (31) has shown that family web-based nutrition interventions can modestly improve dairy product intakes in the short term, highlighting the potential of structured family-based strategies in promoting healthier dietary behaviors. Past research highlighted the role of family meals in shaping healthy eating habits, noting that children who eat with parents are twice as likely to consume five servings of fruits and vegetables daily (32). Moderate restrictions and encouragement (fruit intake OR = 1.357, vegetable intake OR = 1.335) positively influence dietary behavior (33). One previously emphasized the importance of family-based lifestyle interventions in fostering sustainable behavior changes and self-regulation in children with overweight and obesity (34), further underscoring the value of family-centered approaches in improving nutritional outcomes.

Child incentives, social marketing techniques, and local stakeholder collaboration significantly increased intervention effectiveness. Some studies have noted that successful interventions often included setting family-based goals, modifying the home food environment, and providing practical application components (27, 28). We incorporated these elements into our intervention design.

The study findings highlight the multifactorial nature of childhood nutrition and obesity. The observed influence of family-level factors is consistent with prior evidence emphasizing parental dietary practices and feeding styles (20, 32, 33). The associations of sugar-sweetened beverage intake and screen exposure with higher BMIZ are also aligned with previous reports (25, 26). The intervention-related improvements, particularly among children with overweight/obesity, support the potential value of comprehensive family-involved strategies (27–31, 34). Future work should prioritize longer follow-up and broader socioeconomic sampling to test whether these short-term improvements can be sustained.

### 5.1 Limitations

This study has several limitations that should be considered when interpreting the findings. First, the intervention component employed a one-group pre-post design without a concurrent control group, which precludes causal inference. Observed improvements in BMI and NQ-P scores may be attributable to natural growth and developmental changes, seasonal variations in diet and physical activity, regression to the mean, or the Hawthorne effect rather than the intervention itself. Future studies should adopt randomized controlled trial designs with waitlist or usual-care control groups. Second, the 163 intervention participants were recruited through voluntary opt-in, which may introduce selection bias, as these parents may have been more health-conscious or motivated than non-participants. Although baseline characteristics did not differ significantly between intervention participants and non-participants (Supplementary Table 7), unmeasured confounders cannot be excluded. Third, the intervention lasted only three months, which is insufficient to establish sustained behavioral change. For childhood obesity interventions, long-term follow-up of 6–12 months or longer is typically required to demonstrate habit formation and maintenance. Fourth, all dietary data were parent-reported through the NQ-P questionnaire, which is subject to recall bias and social desirability bias. Future studies should consider triangulating self-reported data with objective measures such as 24-h dietary recalls, direct observation of eating behaviors, or biomarker assessments. Fifth, the sample was restricted to kindergartens in Minhang District, Shanghai, a relatively affluent urban area with high parental education levels, limiting the generalizability of findings to other socioeconomic contexts or rural settings. Sixth, no formal intervention fidelity checks were conducted, and compliance with the punch-card program varied across participants. Finally, the analysis primarily relied on bivariate correlations; although we conducted supplementary multivariable regression analyses adjusting for key confounders, residual confounding from unmeasured variables remains possible.

## 6 Conclusion

The study demonstrates that parental involvement in dietary responsibility, frequent family meals, and outdoor exercise positively correlate with higher NQ-P scores. The targeted intervention was associated with reductions in overweight/obesity prevalence and improvements in dietary behaviors, particularly in the balance and moderation domains. The intervention stabilized BMI in normal-weight children and improved outcomes in underweight and overweight subgroups. These results underscore the importance of tailored, family-based strategies to address childhood malnutrition and obesity. Future research should expand to diverse populations and incorporate long-term follow-ups to validate the sustainability of these interventions.

## Data Availability

The original contributions presented in the study are included in the article/Supplementary material, further inquiries can be directed to the corresponding author.
