# Supplementary material for: The correlation between nutrition index scores and nutrition status of preschool children in a district of Shanghai
Source: Front Nutr. 2026 Apr 13;13:1746871. doi: 10.3389/fnut.2026.1746871 (PMC13111329; doi:10.3389/fnut.2026.1746871)
Supplement: Supplementary file 1 [file Table_1.docx]

**Supplementary Materials**

**Supplementary Methods**

**Data Quality Control**

Questionnaire data were directly exported from the online survey platform backend system. BMI Z-scores were calculated independently by two researchers using WHO growth reference standards, with 100% double verification and cross-checking to ensure calculation accuracy.

**Sensitivity Analysis Exclusion Criteria**

Children who reported obvious symptoms of discomfort in the past month (n=129, 7.2% of baseline sample) were excluded from sensitivity analysis. The remaining analytical sample comprised 1,665 children for robustness testing of primary findings.

**Supplementary Table 1: Distribution of NQ-P Scores by Gender and Age Groups (n=1,794)**

| **Variables** | **NQ-P total score** | **Balance domain** | **Moderation**  **domain** | **Environmental domain** |
| --- | --- | --- | --- | --- |
| **Gender** |  |  |  |  |
| Boy (*n* = 950) | 67.42±8.68 | 28.22±5.49 | 46.36±13.38 | 38.49±6.14 |
| Girls (*n* = 844) | 67.40±8.64 | 28.28±5.25 | 45.66±13.91 | 38.89±7.51 |
| *P* | >0.05 | >0.05 | >0.05 | >0.05 |
| **Age** |  |  |  |  |
| 2 ~ <3 (*n* = 2) | 74.55±1.91 | 35.65±4.45 | 57.13±25.28 | 39.30±2.40 |
| ~ <4 (*n* = 183) | 68.11±8.35 | 27.95±5.09 | 47.05±14.40 | 38.51±6.30 |
| ~ <5 (*n* = 523) | 67.00±8.77 | 28.25±5.05 | 45.97±14.32 | 38.63±8.51 |
| ~ <6 (*n* = 584) | 67.45±8.79 | 28.07±5.69 | 46.15±13.17 | 38.38±5.90 |
| ~ <7 (*n* = 499) | 67.53±8.54 | 28.54±5.43 | 45.58±13.15 | 39.14±5.99 |
| ~ <8 (*n* = 3) | 63.73±3.09 | 26.77±3.45 | 41.17±2.52 | 37.60±3.03 |
| *P* | >0.05 | >0.05 | >0.05 | >0.05 |

**Supplementary Table 2：Post-hoc Analysis of NQ-P Scores by BMIZ Groups (n=1,794)**

| **Dependent Variable** | **(I) BMIZ** | **(J) BMIZ** | **Mean difference (I-J)** |
| --- | --- | --- | --- |
| NQ-P total score | 1～3 | 0 | 1.39 ** |
|  |  | -3～-1 | -0.87 |
|  | 0 | -3～-1 | 0.52 |
| Balance **domain** | 1～3 | 0 | 0.62 * |
|  |  | -3～-1 | -0.14 |
|  | 0 | -3～-1 | 0.49 |
| Moderation domain | 1～3 | 0 | 2.47 *** |
|  |  | -3～-1 | 3.41 *** |
|  | 0 | -3～-1 | -0.94 |
| Environmental domain | 1～3 | 0 | 0.60 |
|  |  | -3～-1 | 0.36 |
|  | 0 | -3～-1 | -0.25 |

Note:* *P*<0.05, statistically significant difference；***P*<0.01, statistically significant difference；*** *P*<0.001, statistically significant difference

**Supplementary Table 3: NQ-P Score Differences by Physical Development Groups (n=1,794)**

| **Physical development of children** | **NQ-P total score** | **Balance domain** | **Moderation**  **domain** | **Environmental domain** |
| --- | --- | --- | --- | --- |
| 3 (*n* = 46) | 65.95±8.24 | 26.53±5.32 | 46.29±14.59 | 38.47±4.52 |
| 2 (*n* = 120) | 66.27±8.36 | 27.72±6.20 | 45.51±13.60 | 39.01±6.82 |
| 1 (*n* = 291) | 66.62±8.59 | 28.14±5.56 | 43.07±12.10 | 39.22±5.77 |
| 0 (*n* = 1051) | 67.85±8.66 | 28.48±5.08 | 46.51±13.65 | 38.48±5.89 |
| -1 (*n* = 191) | 68.09±8.39 | 28.06±5.82 | 47.38±12.93 | 38.37±6.49 |
| -2 (*n* = 62) | 64.75±10.02 | 27.90±5.72 | 45.69±12.44 | 38.47±6.51 |
| -3 (*n* = 36) | 67.78±8.63 | 27.83±6.15 | 50.83±23.22 | 41.07±23.89 |
| *P* | <0.05 | >0.05 | <0.001 | >0.05 |

**Supplementary Table 4: Sensitivity Analysis - Distribution of NQ-P Scores by Gender and Age Groups (n=1,665)**

| **Variables** | **NQ-P total score** | Balance domain | **Moderation**  **domain** | Environmental domain |
| --- | --- | --- | --- | --- |
| **Gender** |  |  |  |  |
| Boy (n = 869) | 67.56±8.57 | 28.42±5.47 | 45.93±13.41 | 38.53±5.93 |
| Girls (n = 796) | 67.51±8.62 | 28.18±5.23 | 45.96±13.71 | 39.17±7.66 |
| *P* | 0.899 | 0.364 | 0.963 | 0.056 |
| **Age** |  |  |  |  |
| 2 ~ <3 (n = 2) | 74.55±1.91 | 27.75±2.76 | 66.25±0.00 | 40.60±1.70 |
| ~ <4 (n = 197) | 68.05±8.27 | 28.76±5.78 | 47.29±14.31 | 37.746.12 |
| ~ <5 (n = 493) | 66.93±8.82 | 28.07±5.49 | 46.28±14.15 | 38.568.60 |
| ~ <6 (n = 541) | 67.55±8.67 | 28.40±5.16 | 45.20±12.93 | 39.115.57 |
| ~ <7 (n = 429) | 67.98±8.40 | 28.25±5.49 | 45.83±13.21 | 39.336.17 |
| ~ <8 (n = 3) | 63.73±3.09 | 25.80±4.66 | 41.33±2.25 | 34.803.74 |
| *P* | 0.285 | 0.66 | 0.12 | 0.068 |

**Supplementary Table 5: Sensitivity Analysis - Post-hoc Analysis of NQ-P Scores by BMIZ Groups (n=1,665)**

| **Dependent Variable** | **(I) BMIZ** | **(J) BMIZ** | **Mean difference (I-J)** |
| --- | --- | --- | --- |
| NQ-P total score | 1～3 | 0 | -1.34 |
|  |  | -3～-1 | -0.39 |
|  | 0 | -3～-1 | 0.95 |
| Balance domain | 1～3 | 0 | 0.06 |
|  |  | -3～-1 | 0.96 |
|  | 0 | -3～-1 | 0.90 |
| Moderation domain | 1～3 | 0 | -2.57* |
|  |  | -3～-1 | -2.52 |
|  | 0 | -3～-1 | 0.46 |
| Environmental domain | 1～3 | 0 | 0.07 |
|  |  | -3～-1 | -2.92 |
|  | 0 | -3～-1 | -3.63 |

Note: * *P*<0.05, statistically significant difference

**Supplementary Table 6: Sensitivity Analysis - NQ-P Score Differences by Physical Development Groups (n=1,665)**

| **Physical development of children** | **NQ-P total score** | **Balance domain** | **Moderation**  **domain** | **Environmental domain** |
| --- | --- | --- | --- | --- |
| 3 (*n* =43 ) | 65.86±8.37 | 27.72±4.49 | 45.54±14.15 | 37.32±6.37 |
| 2 (*n* = 113) | 66.26±8.47 | 27.15±6.21 | 44.15±13.16 | 39.34±5.96 |
| 1 (*n* = 272) | 66.79±8.51 | 28.41±5.55 | 43.41±12.30 | 39.19±5.83 |
| 0 (*n* =973 ) | 67.99±8.54 | 28.248±5.11 | 46.52±13.44 | 38.70±5.81 |
| -1(n =171 ) | 68.34±8.34 | 28.51±5.48 | 47.08±13.07 | 39.08±6.04 |
| -2(n = 59) | 64.64±9.86 | 27.00±6.72 | 44.97±13.78 | 37.34±7.28 |
| -3(n =34 ) | 67.99±8.80 | 28.31±5.11 | 52.35±22.45 | 41.46±24.45 |
| *P* | 0.009 | 0.099 | <0.001 | 0.062 |

**Supplementary Table 7: Demographic Characteristics of Intervention Participants (n=156)**

| **Variables** | **Before** | **After** |
| --- | --- | --- |
| **Gender** |  |  |
| Boy | 89 | |
| Girl | 67 | |
| **Age (one year, M±S)** | 4.8±0.44 | 4.98±0.49 |
| 3 ~ <4 | 15(9.6) | 7(4.5) |
| 4 ~ <5 | 73(46.8) | 73(46.8) |
| 5 ~ <6 | 68(43.6) | 73(46.8) |
| 6 ~ <7 |  | 2(1.3) |
| 8 ~ <9 |  | 1(0.6) |
| **BMI (M±S)** | 15.81±3.08 | 16.25±3.78 |
| **BMI Z score (M±S)** | 0.09±0.09 | 0.16±0.09 |
| -3 | 4(2.6) | 4(2.6) |
| -2 | 8(5.1) | 5(3.2) |
| -1 | 16(10.3) | 19(12.2) |
| 0 | 91(58.3) | 92(58.9) |
| 1 | 23(14.7) | 19(12.2) |
| 2 | 6(3.9) | 4(2.6) |
| 3 | 8(5.1) | 13(8.3) |
| **Only child** |  |  |
| yes | 90(57.7) | 90(57.7) |
| no | 66(42.3) | 66(42.3) |
| **Height (cm, M±S)** | 109.51±6.16 | 110.40±6.30 |
| **Weight (kg, M±S)** | 19.03±4.62 | 19.76±4.74 |
| **Maternal education** |  |  |
| High school and below | 1(0.6) | 1(0.6) |
| Associate or undergraduate | 101(64.8) | 103(66.1) |
| Graduate student | 44(28.2) | 44(28.2) |
| Not to be revealed | 10(6.4) | 8(5.1) |
| **Paternal education** |  |  |
| High school and below | 0 | 0 |
| Associate or undergraduate | 93(59.6) | 97(62.2) |
| Graduate student | 54(34.6) | 52(33.3) |
| Not to be revealed | 9(5.8) | 7(4.5) |
| **Whether to be responsible for the diet** |  |  |
| yes | 110(70.5) | 104(66.7) |
| no | 46(29.5) | 52(33.3) |
| **Fill in the relationship between the person and the child** |  |  |
| Mother | 125(80.2) | 128(82.0) |
| Father | 28(17.9) | 24(15.4) |
| Grandparent or maternal grandparent | 3(1.9) | 4(2.6) |
| Other |  |  |
| **Annual household income (10,000 / year)** |  |  |
| <5 | 3(1.9) | 1(0.6) |
| ~ 10 | 3(1.9) | 1(0.6) |
| ~ 15 | 3(1.9) | 2(1.3) |
| ~ 20 | 5(3.2) | 4(2.6) |
| >20 | 79(50.7) | 88(56.4) |
| Not to be revealed | 63(40.4) | 60(38.5) |
| **Number of daily family meals** |  |  |
| ≤3 | 38(24.4) | 38(24.4) |
| 4 | 62(39.7) | 58(37.1) |
| 5 | 35(22.4) | 37(23.7) |
| ≥6 | 21(13.5) | 23(14.8) |
| **Whether the child has obvious symptoms of discomfort in the past month** |  |  |
| is |  |  |
| no |  |  |
| **Parents take the initiative to record their children's eating and drinking.** |  |  |
| Never | 52(33.3) | 47(30.1) |
| Occasionally | 69(44.2) | 65(41.7) |
| Sometimes | 23(14.8) | 27(17.3) |
| Often | 11(7.1) | 12(7.7) |
| All the time | 1(0.6) | 5(3.2) |
| **Extra meal or not** |  |  |
| Extra meals daily | 18(11.5) | 18(11.5) |
| Occasional extra meals | 109(69.9) | 112(71.8) |
| Never extra meals | 29(18.6) | 26(16.7) |
| **Time of day for outdoor exercise** |  |  |
| < 30 minutes | 7(4.5) | 7(4.5) |
| 30 minutes. - An hour | 62(39.7) | 62(39.7) |
| 1 to 2 hours | 64(41.0) | 68(43.6) |
| ≥2 hours | 23(14.8) | 19(12.2) |

**Supplementary Table 8: Changes in NQ-P Scores Before and After Intervention (n=156)**

| **Variables** | **Pre-intervention** | **Post-intervention** |
| --- | --- | --- |
| **NQ-P total score** | 67.51±8.81 | 68.81±9.01* |
| BMIZ = -3 ~ -1 (n = 28) | 67.05±8.18 | 67.21±11.91 |
| BMIZ = 0 (n = 91) | 67.99±9.29 | 69.45±8.27* |
| BMIZ = 1 ~ 3 (n =37) | 66.66±8.14 | 68.45±8.28 |
| **Balance domain** | 28.34±5.77 | 28.79±5.56 |
| BMIZ = -3 ~ -1 (n = 28/28) | 27.59±5.60 | 27.60±7.56 |
| **BMIZ = 0 (**n = 95/95) | 28.49±5.35 | 28.99±4.46 |
| BMIZ = 1 ~ 3 (n = 39/38) | 28.64±6.88 | 29.18±6.24 |
| **Moderation domain** | 18.46±5.32 | 18.93±5.70 |
| BMIZ = -3 ~ -1 (n = 28/28) | 19.04±4.83 | 19.66±5.73 |
| BMIZ = 0 (n = 95/95) | 19.08±5.56 | 19.38 ±5.89 |
| BMIZ = 1 ~ 3 (n = 39/38) | 16.51±4.70 | 17.25±4.98 |
| **Environmental domain** | 19.50±2.52 | 19.67±2.61 |
| BMIZ = -3 ~ -1 (n = 28/28) | 19.78±2.61 | 19.30±2.70 |
| BMIZ = 0 (n = 95/95) | 19.42±2.47 | 19.55±2.69 |
| BMIZ = 1 ~ 3 (n = 39/38) | 19.49±2.62 | 20.24±2.72 |

Note: * *P*<0.05, statistically significant difference
